# Dietary apple polyphenols enhance mitochondrial turnover and respiratory chain enzymes

**DOI:** 10.1113/EP091154

**Published:** 2023-09-01

**Authors:** Yuki Yoshida, Yuki Tamura, Karina Kouzaki, Koichi Nakazato

**Affiliations:** ^1^ Faculty of Medical Science Nippon Sport Science University Tokyo Japan; ^2^ Faculty of Sport Science Nippon Sport Science University Tokyo Japan; ^3^ Graduate School of Health and Sport Science Nippon Sport Science University Tokyo Japan; ^4^ Research Institute for Sport Science Nippon Sport Science University Tokyo Japan; ^5^ Graduate School of Medical and Health Science Nippon Sport Science University Tokyo Japan

**Keywords:** apple polyphenols, mitochondria, mitophagy, rat, TFEB

## Abstract

Previous studies have demonstrated the beneficial effects of apple polyphenol (AP) intake on muscle endurance. Since mitochondria are critical for muscle endurance, we investigated mitochondrial enzyme activity, biogenesis, degradation and protein quality control. Twenty‐four Wistar rats were randomly fed a 5% AP diet (5% AP group, *n* = 8), a 0.5% AP diet (0.5% AP group, *n* = 8), or a control diet (control group, *n* = 8). After a 4‐week feeding period, the expression level of peroxisome proliferator‐activated receptor γ coactivator‐1α, a mitochondrial biosynthetic factor, did not increase, whereas that of transcription factor EB, another regulator of mitochondrial synthesis, significantly increased. Moreover, the mitochondrial count did not differ significantly between the groups. In contrast, mitophagy‐related protein levels were significantly increased. The enzymatic activities of mitochondrial respiratory chain complexes II, III and IV were significantly higher in the AP intake group than in the control group. We conclude that AP feeding increases the activity of respiratory chain complex enzymes in rat skeletal muscles. Moreover, mitochondrial biosynthesis and degradation may have increased in AP‐treated rats.

## Abstract (highlights)

- **What is the central question of this study?**
  Does the administration of apple polyphenols (AP) affect mitochondrial respiratory chain complex enzyme activity, biogenesis, degradation and protein quality control in rat skeletal muscles?
- **What is the main finding and its importance?**
  AP feeding increases respiratory chain complex enzyme activity in rat skeletal muscle. Moreover, AP administration increases transcription factor EB activation, and mitophagy may be enhanced to promote degradation of dysfunctional mitochondria, but mitochondrial protein quality control was not affected.

## 1 INTRODUCTION

Apple (*Malus pumila* Fuji), a member of the family Rosaceae, is the most widely grown fruit crop worldwide. Apples, particularly unripe apple peel and seeds, contain various polyphenols (Francini & Sebastiani, 2013). The antioxidant activity of polyphenols isolated from apples is the strongest among all fruits (Eberhardt et al., 2000). Apple polyphenols (AP) comprise procyanidin dimers to pentadecamers (45%), phenolic acids (25%, mainly chlorogenic acid), monomeric flavan‐3‐ols (15%, mainly catechin), phloretin glycosides (10%, mainly phloridzin) and other components (5%, mainly quercetin glycosides). This composition is typical of natural apple polyphenols extracted from unripe apples (Hammerstone et al., 2000; Kanda et al., 1998; Ohnishi‐Kameyama et al., 1997; Shoji et al., 2000; Vinson et al., 2001), and was used in this study. Procyanidins are important components comprising (+)‐catechin and (−)‐epicatechin units, which are widely found as secondary metabolites in plants (Hammerstone et al., 2000; Vinson et al., 2001). Both in vitro and in vivo studies have demonstrated that AP and purified procyanidins have various biological functions, including antioxidant activity (Eberhardt et al., 2000; Lee et al., 2003; Pearson et al., 1999), immune function modulation (Kanda et al., 1998), adipose tissue mass reduction (Nakazato et al., 2006), prevention of muscle injury (Nakazato et al., 2010) and increased energy consumption (Tamura et al., 2020).

Nakazato et al. (2007) and Mizunoya et al. (2015) evaluated the isometric tetanic torque of the rat ankle joint and reported improved muscle endurance in animals fed with 5% AP. Furthermore, the relative amount of MyHC IIb was significantly reduced, whereas MyHC IIx content was increased in the gastrocnemius muscles of 5% AP‐fed rats. These findings suggested that fast‐to‐slow MyHC changes occurred in AP‐fed rats. Additionally, oral AP administration increased skeletal muscle capillaries in Wistar rats (Yoshida et al., 2018).

During the evolution of organisms, mitochondria have been conserved organelles that convert energy substrates into ATP via aerobic respiration (Gray et al., 2001; Searcy, 2003). Endurance training increases the activity of oxidative enzymes in the rat skeletal muscle mitochondria (Holloszy, 1967). The expression of mitochondrial‐related genes is increased by endurance training (Murakami et al., 1994). Therefore, increased mitochondrial content and function are essential for endurance capacity (Cunningham, 1990; Maughan & Leiper, 1983). Mitochondrial protein quality control by molecular chaperones and mitophagy, the selective dysfunctional mitochondrial degradation by autophagy, is also important for maintaining and improving mitochondrial function (Jadiya & Tomar, 2020).

Although APs are candidate compounds for improving muscle endurance (Mizunoya et al., 2015; Nakazato et al., 2007), the effect of APs on the mitochondria remains unclear. In this study, we focused on whether dietary APs alter mitochondrial respiratory chain complex enzyme activity, biogenesis and degradation, and quality control.

## 2 METHODS

### 2.1 Ethical approval

This study was approved by the Animal Experimental Committee of the Nippon Sport Science University (No. 010‐A01). The authors have read, and all experiments complied with, the policies and regulations of the Fundamental Guidelines for Proper Conduct of Animal Experiments and Related Activities in Academic Research Institutions published by the Ministry of Education, Culture, Sports, Science and Technology, Japan. All experiments conformed to the principles and standards for reporting animal experiments in *Experimental Physiology* (Grundy, 2015). All measures were taken to minimize animal suffering.

### 2.2 Animals, experimental diets

Twenty‐four 9‐week‐old male Wistar rats were obtained from Japan Clea (Tokyo, Japan) and maintained at 23 ± 1°C under a 12:12 h light–dark cycle. All animals were fed laboratory chow (CE7; Clea) for 1 week and then fed one of the following three diets: AIN‐93M‐based normal diet supplemented with 5% AP (5% AP group, *n* = 8), AIN‐93M‐based normal diet supplemented with 0.5% AP (0.5% AP group, *n* = 8) or AIN‐93M‐based normal diet (control group, *n* = 8). Animals were weighed, and then animals of similar weights were randomly allocated to one of the three groups to ensure each group's starting weight was similar. Rats were housed individually per cage and had free access to drinking water at all times. The dietary composition of each group is shown in Table 1. AP was provided by Asahi Breweries, Ltd. (Tokyo, Japan). All animals were maintained on diets for 4 weeks, and their weight and food intake were recorded every 2 days throughout the experimental period. The animals were anaesthetized using an overdose of isoflurane inhalation (Pfizer, New York, NY USA), and after confirming respiratory arrest, killed by blood removal from the abdominal aorta. The medial and lateral gastrocnemius muscles were also collected. After weighing, the tissues were immediately frozen in liquid nitrogen and stored at −80°C for biochemical analyses.

**TABLE 1:** Control and apple polyphenol (AP) diet composition.

		Diet (g (%))	
Ingredients	Control	0.5% AP	5% AP
Casein	14.0 (14.0)	14.0 (13.93)	14.0 (13.33)
Cornstarch	46.5692 (46.5692)	46.5692 (46.33751)	46.5692 (44.35162)
α‐Cornstarch	15.5 (15.5)	15.5 (15.42)	15.5 (14.76)
Sucrose	10.0 (10.0)	10.0 (9.95)	10.0 (9.52)
Soybean oil	4.0 (4.0)	4.0 (3.98)	4.0 (3.81)
Cellulose	5.0 (5.0)	5.0 (4.98)	5.0 (4.76)
Mineral mixture	3.5 (3.5)	3.5 (3.48)	3.5 (3.33)
Vitamin mixture	1.0 (1.0)	1.0 (1.00)	1.0 (0.95)
l‐cystine	0.18 (0.18)	0.18 (0.179)	0.18 (0.171)
Choline(bitartrate)	0.25 (0.25)	0.25 (0.249)	0.25 (0.238)
t‐Butylhydroquinone	0.0008 (0.0008)	0.0008 (0.00080)	0.0008 (0.00076)
AP	0 (0)	0.5 (0.50)	5 (4.8)

AIN mineral and vitamin mixtures were obtained from Oriental Yeast Co. (Tokyo, Japan). The composition of the control diet was based on AIN93M as reported by the American Institute of Nutrition. The 0.5% AP and 5% AP diets were modified from the control diet. Abbreviation: AP, apple polyphenols.

### 2.3 Western blot analysis

Western blot analysis was performed to investigate the effects of AP on the mitochondria of the gastrocnemius muscle. Frozen tissues were homogenized using radioimmunoprecipitation assay (RIPA) buffer (Thermo Fisher Scientific, Waltham, MA, USA) containing protease inhibitors (Complete Mini, Roche Diagnostics, Basel, Switzerland), and total protein concentrations were measured using the bicinchoninic acid (BCA) method (Pierce BCA Protein Assay Kit, Thermo Fisher Scientific). Equal protein amounts (10−20 μg) were separated using SDS‐PAGE and transferred on polyvinylidene fluoride membranes (IPVH00010, Merck Millipore, Burlington, MA, USA). Protein transfer was confirmed by staining with Ponceau S (33427.01, SERVA Electrophoresis, Heidelberg, Germany). The transferred membranes were blocked with a blocking reagent (NYPBR01, Toyobo Company, Ltd, Osaka, Japan) at room temperature for 1 h with gentle shaking. The membranes were then incubated with primary antibodies diluted in Reagent 1 (NKB‐101, Toyobo) at room temperature for 1 h with gentle shaking.

The primary antibodies used in this study were: total OXPHOS (1:5000; cat. no. ab 110413, Abcam, Cambridge, UK), peroxisome proliferator activated receptor γ coactivator‐1 (PGC‐1) C‐terminus (777–797) (1:1000; cat. no. 516557, Millipore, Darmstadt, Germany), transcription factor EB (TFEB) (1:1000; cat. no. 32361, Cell Signaling Technology, Danvers, MA, USA), transcription factor binding to IGHM enhancer 3 (TFE3) (1:1000; cat. no. 14779, Cell Signaling Technology), cytochrome *c* (1:1000; cat. no. 12959, Cell Signaling Technology), voltage‐dependent anion channel (VDAC) (1:1000; cat. no. 12454, Cell Signaling Technology), heat shock protein 60 (HSP60) (1:10000; cat. no. ADI‐SPA‐806, Enzo Life Sciences, Farmingdale, NY, USA), heat shock protein 70 (mtHSP70) (1:5000; cat. no. ALX‐804‐077‐R100, Enzo Life Sciences), caseinolytic mitochondrial matrix peptidase proteolytic subunit (ClpP) (1:5000; cat. no. HPA010649, Sigma‐Aldrich, MO, USA), ubiquitin (P37) (1:1000; cat. no. 58395, Cell Signaling Technology), microtubule‐associated protein 1 light chain 3B (LC3B) (1:1000; cat. no. 2775, Cell Signaling Technology), histone H3 (1:10,000, cat. no. 4499, Cell Signaling Technology), cytochrome *c* oxidase subunit IV (COX IV) (1:10,000, cat. no. 4844, Cell Signaling Technology) and heat shock cognate (HSC70) (1:10,000, cat. no. ADI‐SPA‐815, Enzo Life Sciences).

After washing with Tris‐buffered saline containing 0.01% Tween 20 (T9142, Takara Bio, Shiga, Japan) (TBST), the membranes were incubated at room temperature for 1 h with horseradish peroxidase‐labeled anti‐rabbit or anti‐mouse immunoglobulin (Cell Signaling Technology) diluted in reagent 2 (NKB‐101, Toyobo) as the secondary antibody. The blots were washed with TBST, and the proteins were detected with chemiluminescence reagents (Super Signal West Pico Chemiluminescent Substrate, Thermo Fisher Scientific), followed by visualization with a CCD imager (ChemiDoc XRS Plus, 170‐8071, Bio‐Rad Laboratories, Hercules, CA, USA) and quantification using Image Lab software (Bio‐Rad). Ponceau S staining was used to normalize loading, which was then normalized to the control group (protein expression/Ponceau S [relative to Control group]).

### 2.4 Nuclear fractionation

Nuclear and mitochondrial fractions were obtained by differential centrifugation with minor modifications as previously described (Dimauro et al., 2012; Wakabayashi et al., 2020). All buffers had the same composition, as described by Wakabayashi et al. (2020).

Frozen tissues were ground using a homogenizer. Immediately after grinding, the gastrocnemius muscles were placed in a homogenization buffer and homogenized for 10 strokes using a Potter glass homogenizer. The tissue homogenates were centrifuged at 500 *g* for 10 min at 4°C, and the supernatants (Solution S) were used to subsequently isolate the mitochondrial fraction (described in ‘Mitochondrial fractionation’).

Briefly, the obtained pellets were washed three times with a washing buffer. The pellets were then suspended in a buffer and centrifuged at 1000 *g* for 15 min at 4°C. New pellets were resuspended in nuclear extraction buffer and incubated on ice for 30 min, with vortexing every 5 min. After incubation, the suspensions were passed through an 18‐gauge needle 30 times. The lysates were centrifuged at 9000 *g* for 30 min at 4°C and the final supernatants were collected. Protein concentrations were determined by the BCA method (Pierce BCA Protein Assay Kit; Thermo Fisher Scientific). The lysates were analysed by western blotting.

### 2.5 Mitochondrial fractionation

Mitochondrial fractions were extracted from Solution S by centrifugation as previously described, with minor modifications (Fisher‐Wellman et al., 2018; Wakabayashi et al., 2020). Briefly, Solution S was centrifuged at 10,000 *g* for 10 min at 4°C to obtain the mitochondrial pellets, which were washed in buffer (50 mM MOPS, 100 mM KCl, 1 mM EGTA, and 5 mM MgSO_4_) and resuspended in buffer (10 mM Tris, 30 mM KCl, 10 mM KH_2_PO_4_, 5 mM MgCl_2_, 1 mM EGTA, and 2.5 g/l BSA, pH 7.2). For western blotting, mitochondrial fractions were re‐pelleted and suspended in RIPA buffer. The protein concentration was determined using the BCA method.

### 2.6 Measurements of mitochondrial content

The number of mitochondria was measured from several perspectives using western blot analysis and quantitative real‐time PCR. The protein expression levels of cytochrome *c*, VDAC, and OXPHOS complexes, which comprise the mitochondrial respiratory chain complex (CI‐NDUFB8, CII‐SDHB, CIV‐MTCO1, CIII‐UQCR2 and CV‐ATP5A), were examined.

#### 2.6.1 PCR analysis of mitochondrial DNA

The mitochondrial DNA (mtDNA) and genomic nuclear DNA (nDNA) copy numbers were performed by quantitative real‐time PCR, and the nDNA to mtDNA ratio was calculated. Total RNA was extracted from the gastrocnemius muscle using the DNeasy Blood & Tissue Kit (Qiagen, Shanghai, China) following the manufacturer's instructions. Frozen and homogenized tissues were placed in a tissue lysis (ATL) buffer for DNA extraction (ATL and proteinase K) and incubated at 56°C until the tissues were completely lysed. To obtain RNA‐free genomic DNA, 4 μl 100 mg/ml RNase A was added and incubated for 2 min at room temperature. Genomic DNA was precipitated using AL buffer and 100% ethanol, and the samples were loaded onto DNeasy mini spin columns. The columns were washed with Buffer AW and centrifuged for 3 min at 20,000 *g* to dry the DNeasy membranes. After centrifugation, purified genomic DNA was eluted with nuclease‐free water. The concentration of the purified DNA was determined by spectrophotometric absorption (NanoDrop, Kanto Chemical Co., Inc., Tokyo, Japan) at 230, 260 and 280 nm, and the quality was calculated as the *A*_260_/*A*_230_ and *A*_260_/*A*_280_ ratios and used as a PCR template. After purifying the genomic DNA, real‐time quantitative PCR amplification was performed using a Thunderbird SYBR qPCR Mix kit (Toyobo). Next, 10 ng of purified DNA was used to amplify the mtDNA and nDNA markers with a thermal cycler (CFX96 Real‐Time System, Bio‐Rad) simultaneously on the same plate using the following programme: 30 s at 95°C, 5 s at 95°C and 60 s at 60°C for 40 cycles. mtDNA was amplified using primers specific for the mitochondrial 16S rRNA gene and mitochondrial tRNA, and the mitochondrial DNA copy number was normalized to the nDNA copy number by amplifying glyceraldehyde‐3‐phosphate dehydrogenase (GAPDH) and β‐actin genes (Abdullaev et al., 2020; Sethumadhavan et al., 2012; Xie et al., 2014). The primers used are listed in Table 2.

**TABLE 2:** List of primers used in RT‐PCR.

	Forward primer (5′−3′)	bps	Reverse primer (5′−3′)	bps
mt–tRNA	AATGGTTCGTTTGTTCAACGATT	23	AGAAACCGACCTGGATTGTC	21
GAPDH	TGGCCTCCAAGGAGTAAGAAAC	22	GGCTCTCTCCTTGCTCTCAGTTAC	24
mt−16S rRNA	CAATTCTCCTAGCACAAGTG	20	CCCAACCGAAATTTTTTAGTTC	22
β‐Actin	CTATGTTGCCCTAGACTTCGAGC	23	TTGCCGATAGTGATGACCTGAC	22

### 2.7 Mitochondrial enzyme activity

Mitochondrial enzyme activity was measured as previously described with minor modifications (Spinazzi et al., 2012; Wakabayashi et al., 2020). All buffers had the same composition, as described by Wakabayashi et al. (2020). After removing any visible fat or connective tissue, the frozen muscle was ground into small fragments. The muscle was homogenized using an ultrasonic wave in the ice‐cold buffer. Tissue homogenates were centrifuged at 1500 *g* for 5 min at 4°C and the supernatants were collected. The concentration of each purified protein was determined by the BCA method and adjusted to 2.0 μg/μl.

#### 2.7.1 Citrate synthase

A protein suspension (10 μg) was mixed with the reaction buffer in a 96‐well plate. Changes in absorbance per minute at 412 nm were calculated.

#### 2.7.2 Complex I

Protein suspension (10 μg) was mixed with reaction buffer in a 96‐well plate. Changes in absorbance per minute at 340 nm were calculated. Rotenone‐sensitive enzyme activity (rotenone absence and presence) was regarded as complex I activity.

#### 2.7.3 Complex II

Protein suspension (10 μg) was mixed with reaction buffer in a 96‐well plate. Changes in absorbance per minute at 600 nm were calculated.

#### 2.7.4 Complex I+III

Protein suspension (10 μg) was mixed with reaction buffer in a 96‐well plate. Changes in absorbance per minute at 550 nm were calculated.

#### 2.7.5 Complex II+III

Protein suspension (10 μg) was mixed with reaction buffer in a 96‐well plate. Changes in absorbance per minute at 550 nm were calculated.

#### 2.7.6 Complex IV

Protein suspension (10 μg) was mixed with reaction buffer in a 96‐well plate. Changes in absorbance per minute at 550 nm were calculated.

### 2.8 Statistical analysis

All values are presented as means ± standard deviation (SD). The Shapiro–Wilk test was performed, and all data were found to be normal. One‐way analysis of variance (ANOVA) and Fisher's least significant difference (LSD) test were performed to compare means among the three groups. This multiple‐comparison test is effective for comparing three groups (Hayter, 1986). Statistical significance was defined as *P* < 0.05, and a trend was defined as *P* < 0.10. Statistical analyses were performed on a Windows computer with a statistical software package (SPSS version 26; IBM Corp., Armonk, NY, USA).

## 3 RESULTS

### 3.1 Food consumption, body weight and tissue weight

**FIGURE 1:**
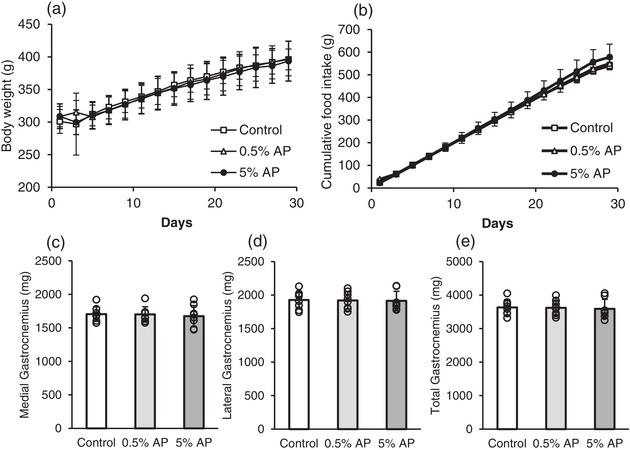
Cumulative food intake, body weight and tissue weight. Effects of dietary AP on (a) body weight, (b) cumulative food intake, and (c–e) muscle weight. Data are presented as means ± SD. Statistical significance was evaluated by one‐way ANOVA with Fisher's protected least significant difference test. Control, AIN‐93M‐based normal diet; AP, AIN‐93M‐based normal diet supplemented with apple polyphenols; *n* = 8 for each group.

To investigate the effects of AP feeding, the amount of food consumed and body and tissue weights were measured. Average body weight was not significantly different among the groups (Figure 1a). Moreover, the cumulative food intake was not significantly different (Figure 1b). The medial and lateral gastrocnemius muscle weights were not significantly different among the control, 0.5% AP and 5% AP groups (Figure 1c–e). These tendencies are similar to those previously reported (Yoshida et al., 2018).

### 3.2 Cytochrome *c* protein expression level and citrate synthase activity

**FIGURE 2:**
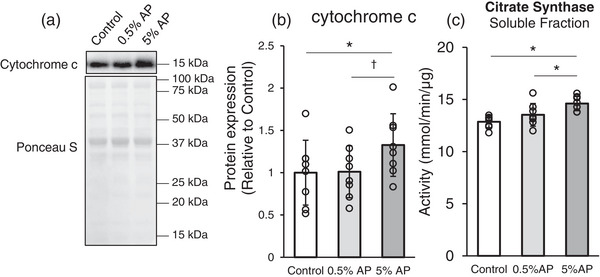
Effect of dietary AP on cytochrome *c* and citrate synthase activity in gastrocnemius. (a) Representative western blot. (b) Western blot analysis of cytochrome *c*. Protein levels was normalized to the ponceau S and then normalized to the control group (protein expression/Ponceau S (relative to Control group). (c) Activity of citrate synthase. Data are presented as means ± SD. Statistical significance was evaluated by one‐way ANOVA with Fisher's protected least significant difference test. Statistical significance was expressed by *P < 0.05 and trend was expressed by †*P* < 0.10. Control, AIN‐93M‐based normal diet; AP, AIN‐93M‐based normal diet supplemented with apple polyphenols; *n* = 8 for each group.

To evaluate the effect of AP on skeletal muscle mitochondria, we first analysed cytochrome *c* protein expression levels and citrate synthase activity. Cytochrome *c* protein levels in the 5% AP group were significantly higher and tended to be higher than those in the control and 0.5% AP groups, respectively (Figure 2b). Citrate synthase activity in the 5% AP group was significantly higher than those in the control and 0.5% AP groups (Figure 2c).

### 3.3 Mitochondrial biogenesis

**FIGURE 3:**
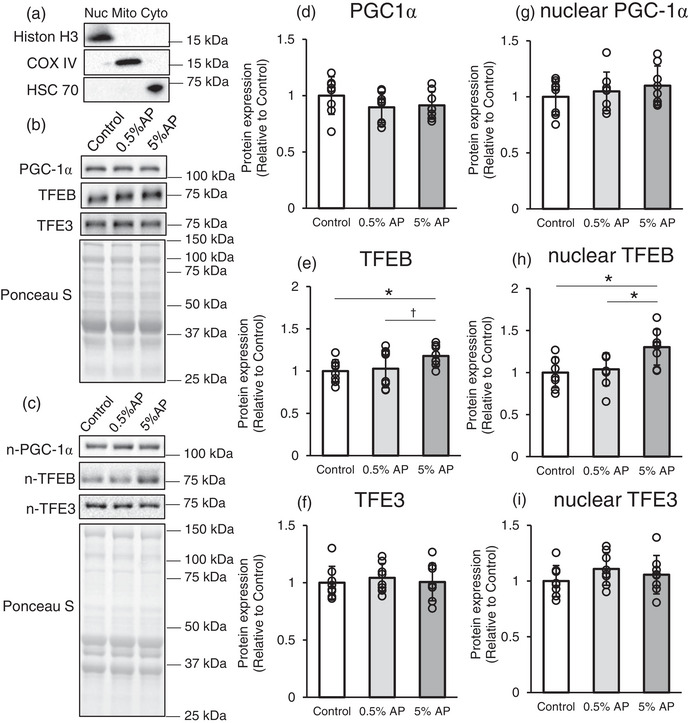
Effect of dietary AP on the mitochondrial biogenesis and related proteins in gastrocnemius. (a) The purity of the fractions was confirmed using nuclear (Nuc; histone H3), mitochondrial (Mito; COX IV), and cytosolic (Cyto; HSC70) marker proteins. (b,c) Representative western blot. (d–f) Protein levels of PGC‐1α, TFEB and TFE3, respectively. (g–i) Protein levels of nuclear fraction of PGC‐1α, TFEB, and TFE3, respectively. Protein levels were normalized to the ponceau S and then normalized to the control group (protein expression/Ponceau S [relative to Control group]). Data are presented as means ± SD. Statistical significance was evaluated by one‐way ANOVA with Fisher's protected least significant difference test. Statistical significance was expressed by *P < 0.05 and trend was expressed by †*P* < 0.10. Control, AIN‐93M‐based normal diet; AP, AIN‐93M‐based normal diet supplemented with apple polyphenols; *n* = 8 for each group.

As AP supplementation increases citrate synthase activity, it may also increase mitochondrial biogenesis. Therefore, we investigated the expression of the peroxisome proliferator‐activated receptor γ coactivator‐1α (PGC‐1α) protein, a key factor in mitochondrial biogenesis (Lin et al., 2002; Russell et al., 2004). However, no significant differences were observed between the control, 0.5% AP and 5% AP groups (Figure 3d).

Because transcription factor EB (TFEB) is involved in mitochondrial biogenesis (Mansueto et al., 2017), we focused on TFEB and its related protein, transcription factor binding to IGHM enhancer 3 (TFE3). The level of TFEB protein was significantly higher in the 5% AP group than in the control group (Figure 3e). However, the TFE3 levels were not significantly different between the groups (Figure 3f). The active forms of these transcriptional regulators translocate to the nucleus and bind to the transcriptional regulatory region of DNA to enhance target gene expression levels. Therefore, we prepared the nuclear, mitochondrial and cytosolic fractions by differential centrifugation. The purity of the fractions was determined by measuring the expression of compartment‐specific proteins (nuclear: histone H3; mitochondrial: COX IV; and cytosolic: HSC70; Figure 3a). PGC‐1α, TFEB and TFE3 protein expression levels were measured using the nuclear fraction, and the results showed that PGC‐1α and TFE3 protein levels were not significantly different between the groups (Figure 3g,i), whereas TFEB protein levels were substantially increased in the 5% AP group (Figure 3h).

### 3.4 Mitochondrial content

**FIGURE 4:**
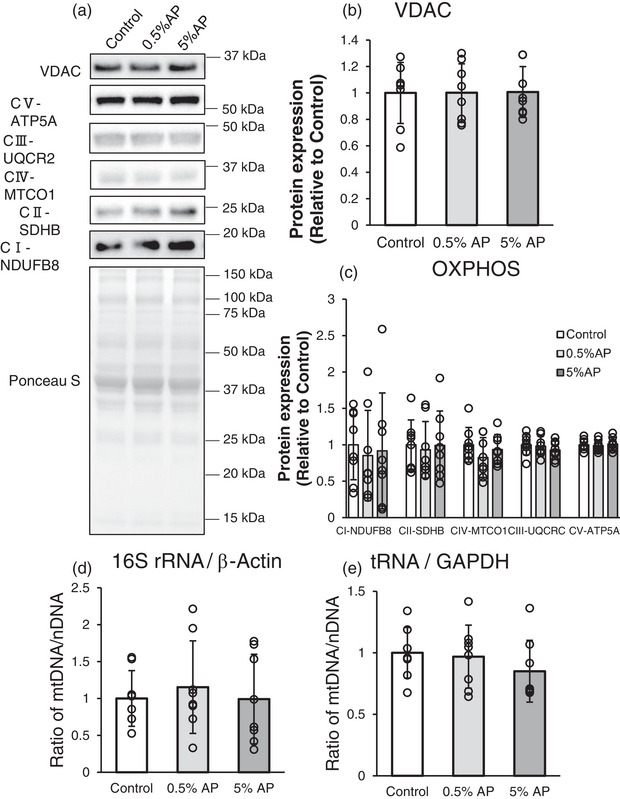
Effect of dietary AP on the mitochondrial contents in gastrocnemius. (a) Representative western blot. (b) Protein level of VDAC. (c) Protein levels of CI‐NDUFB8, CII‐SDHB, CIV‐MTCO1, CIII‐UQCR2 and CV‐ATP5A. Protein levels were normalized to the ponceau S and then normalized to the control group (protein expression/Ponceau S [relative to Control group]). (d,e) Change in the ratio of mtDNA to nDNA compared to the control. (d) 16S rRNA/β‐actin ratio. (e) tRNA/GAPDH ratio. Data are presented as mean ± SD. Statistical significance was evaluated by one‐way ANOVA with Fisher's protected least significant difference test. Control, AIN‐93M‐based normal diet; AP, AIN‐93M‐based normal diet supplemented with apple polyphenols; *n* = 8 for each group.

As the number of active forms of TFEB increased, we hypothesized that the mitochondrial count would have increased. Therefore, in this study, we investigated the protein expression level of VDAC, an ion channel that opens in response to changes in the membrane potential of the outer mitochondrial membrane, as a measure of mitochondrial count, which was not significantly different (Figure 4b). To further investigate the mitochondrial levels, we measured the expression of OXPHOS subunit proteins: CI‐NDUFB8 (complex I; NADH dehydrogenase [ubiquinone] 1 β subcomplex subunit 8), CII‐SDHB (complex II; succinate dehydrogenase complex iron–sulfur subunit B), CIV‐MTCO1 (complex IV; mitochondrially encoded cytochrome *c* oxidase I [COX1]), CIII‐UQCR2 (complex III; cytochrome *b*–*c*_1_ complex subunit 2) and CV‐ATP5A (complex V; ATP synthase α‐subunit), which are individual subunits of the electron transport chain complex. However, the expression levels of these proteins did not differ significantly among the three groups (Figure 4c).

Subsequently, we investigated the mtDNA copy number using real‐time PCR. The mtDNA‐to‐nDNA ratio was not significantly different between the 16S rRNA and β‐actin primer sets and between the tRNA and GAPDH primer sets (Figure 4d,e). Furthermore, real‐time PCR was performed with combinations of 16S rRNA and GAPDH primer sets and tRNA and β‐actin primer sets and the amount of mtDNA relative to nDNA was calculated; however, no significant difference was observed (data not shown).

### 3.5 Mitochondrial protein quality control

The mitochondria contain organelle‐specific molecular chaperones and proteases. To adapt to protein structural abnormalities caused by stress, such as changes in the external environment and metabolism, mitochondria have a protein quality control mechanism known as the mitochondrial unfolded protein response (UPRmt) that regulates gene expression (Haynes & Ron, 2010).

**FIGURE 5:**
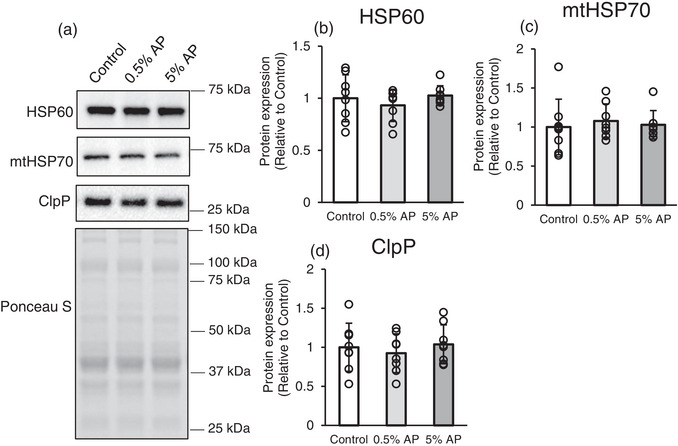
Effect of dietary AP on mitochondrial protein quality control in gastrocnemius. (a) Representative western blot. (b–d) Protein levels of HSP60, mtHSP70 and ClpP, respectively. Protein levels were normalized to the ponceau S and then normalized to the control group (protein expression/Ponceau S [relative to Control group]). Data are presented as means ± SD. Statistical significance was evaluated by one‐way ANOVA with Fisher's protected least significant difference test. Control, AIN‐93M‐based normal diet; AP, AIN‐93M‐based normal diet supplemented with apple polyphenols; *n* = 8 for each group.

This study investigated the expression of heat shock protein 60 (HSP60), mitochondrial heat shock protein 70 (mtHSP70) and caseinolytic mitochondrial matrix peptidase proteolytic subunit (ClpP), which are subunits of ClpXP (caseinolytic mitochondrial matrix peptidase chaperone subunit X (ClpX) and ClpP complex) that reside in the mitochondrial matrix and are encoded by nuclear genes (Neupert & Herrmann, 2007). However, no significant differences were observed in the expression of any of these proteins among the three groups (Figure 5a–d).

### 3.6 Mitophagy

Mitophagy, a mitochondria‐specific autophagy pathway, may play a role in mitochondrial quality control by degrading and recycling excess mitochondria, removing damaged or dysfunctional mitochondria and protecting cells from injury (Youle & Narendra, 2011). It is induced by decreased membrane potential‐mediated mitochondrial dysfunction (Nowikovsky et al., 2007; Priault et al., 2005).

**FIGURE 6:**
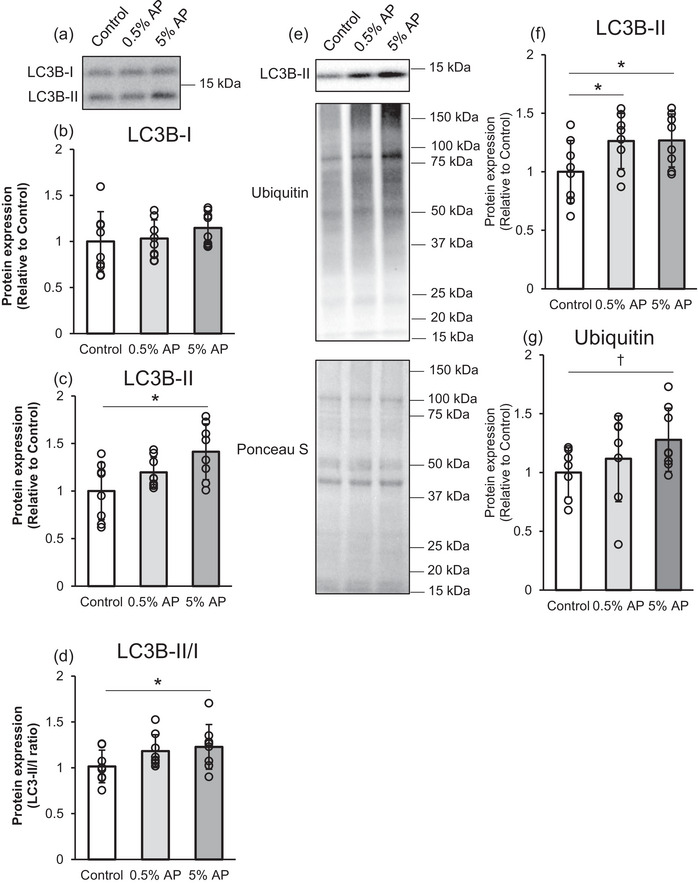
Effect of dietary AP on the mitochondrial autophagy in gastrocnemius. (a) Representative western blot. (b–d) Protein levels of LC3B‐I, LC3B‐II and their ratio in the entire gastrocnemius. (e) Representative western blot. (f) Protein levels of LC3B‐II in the mitochondrial fraction. (g) Protein levels of ubiquitin conjugated protein. Protein levels were normalized to the ponceau S and then normalized to the control group (protein expression/Ponceau S [relative to Control group]). Data are presented as means ± SD. Statistical significance was expressed by *P < 0.05 and trend was expressed by †*P* < 0.10. Control, AIN‐93M‐based normal diet; AP, AIN‐93M‐based normal diet supplemented with apple polyphenols; *n* = 8 for each group.

This study investigated LC3B and ubiquitin‐conjugated proteins as autophagy markers using western blot analysis. LC3B‐II and LC3B‐II/I ratios in the entire gastrocnemius muscle in the 5% AP group were significantly higher than those in the control group (Figure 6c,d). The mitochondrial fraction of LC3B‐II protein in both the 0.5% and 5% AP groups was significantly higher than that in the control group (Figure 6f). Furthermore, the number of ubiquitin‐conjugated proteins in the 5% AP group tended to be higher than that in the control group, although the difference was not statistically significant (Figure 6g).

### 3.7 Enzymatic activity of the mitochondrial respiratory chain complex

To investigate the effects of AP on the skeletal muscle mitochondria, the enzymatic activity of each respiratory chain component (complexes I, II, I + III, II + III and IV) was measured.

**FIGURE 7:**
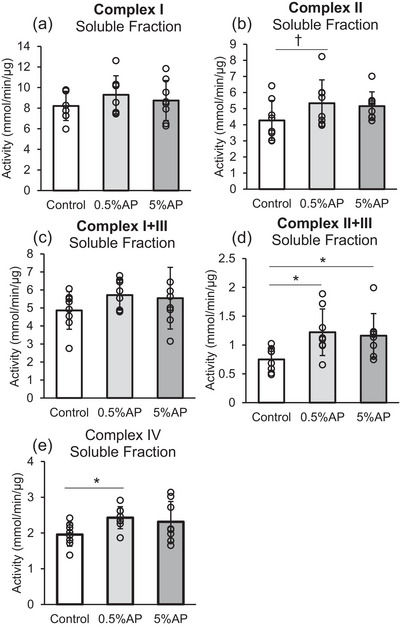
Enzyme activity of mitochondrial respiratory chain complex in the gastrocnemius muscle. (a) Complex I activity, measured by NADH oxidation for rats consuming a control, 0.5% apple polyphenol (AP), and 5% AP diet. (b) Complex II activity, assessed by dichlorophenolindophenol reduction. (c) Complex I + III activity measured by NADH oxidation coupled to cytochrome *c* reduction. (d) Complex II + III activity assayed by succinate oxidation coupled to cytochrome *c* reduction. (e) Complex IV activity, measured by cytochrome *c* oxidation. Data are presented as means ± SD. Statistical significance was evaluated by one‐way ANOVA with Fisher's protected least significant difference test. Statistical significance was expressed by *P < 0.05 and trend was expressed by †*P* < 0.10. Control, AIN‐93M‐based normal diet; AP, AIN‐93M‐based normal diet supplemented with apple polyphenols; *n* = 8 for each group.

The enzymatic activity of the mitochondrial respiratory chain complex II + III in both 0.5% and 5% AP groups was significantly higher than that in the control group (Figure 7d). Moreover, the activity of mitochondrial respiratory chain complex IV in the 0.5% AP group was significantly higher than that in the control group (Figure 7e). Furthermore, the activity of complex II in the 0.5% AP group tended to be higher than that in the control group, although the difference was not statistically significant (Figure 7b).

## 4 DISCUSSION

This study revealed a significant increase in respiratory chain complex enzyme activation in AP‐treated rat skeletal muscles. AP treatment increases TFEB protein expression and nuclear translocation, further enhancing mitochondrial turnover.

Mitochondrial quality and quantity are important factors in determining muscle endurance. In the present study, citrate synthase activity was significantly higher in AP‐treated rats. Since citrate synthase activity correlates with mitochondrial count (Larsen et al., 2012), we considered that the mitochondrial content may have increased. Therefore, we investigated the expression level of PGC‐1α, but no significant difference was observed between the groups. Recently, the existence of a PGC‐1α‐independent mitochondrial regulatory pathway has been demonstrated (Canto et al., 2015; Gomes et al., 2013; Prolla & Denu, 2014). Leick et al. (2008) showed that PGC‐1α is not mandatory for exercise‐induced adaptive gene responses in PGC‐1α knock‐out mouse skeletal muscle. In contrast, TFEB, a well‐known transcription factor that induces lysosomal biogenesis (Sardiello et al., 2009), is also been reported to be involved in mitochondrial biogenesis (Kim et al., 2018). Mansueto et al. (2017) found that TFEB‐knockout mice were exercise‐intolerant, whereas muscle‐specific TFEB activation enhanced physical performance. Additionally, mitochondrial mass and density increased in PGC‐1α knock‐out mice after exercise. Our results indicated that the PGC‐1α protein expression level did not increase, whereas the TFEB expression level in the nuclear fraction increased. Thus, TFEB may be involved in mitochondrial biogenesis. However, several variants of PGC‐1α have been identified (Martínez‐Redondo et al., 2015), and one of these variants was identified in this study. Some effects of TFEB have been reported to be mediated by PGC‐1α (Erlich et al., 2018). The TFEB upregulation observed in this study may be the result of PGC‐1α‐independent pathways, or it may be the result of a PGC‐1α variant that was not detected in this study; thus, further investigation is required.

The mitochondria are continuously exposed to oxidative stress. Accordingly, they possess mechanisms for repairing and degrading mitochondrial DNA and proteins. Mitophagy is the selective removal of dysfunctional mitochondria via autophagy (Choubey et al., 2022). This study used mitochondrial fractions to detect LC3‐II, which was significantly increased in the AP‐treated group. Remarkably, TFEB regulates mitophagy (Pickles et al., 2018; Zhu et al., 2020). Ivankovic et al. (2016) showed that the chemical induction of mitophagy using the uncoupler carbonyl cyanide *m*‐chlorophenylhydrazone increased the amount of TFEB protein and p62 expression decreased in TFEB knockdown cells. Furthermore, TFEB increased mitochondrial biogenesis. Therefore, TFEB is considered an important factor in promoting mitophagy and mitochondrial biogenesis. The mitochondrial count did not change in this study, probably due to the balance between increased degradation by mitophagy and increased biosynthesis.

The most important finding of this study is the increased enzymatic activity of the mitochondrial respiratory chain complex. Enzymatic activity of the respiratory chain complex in the inner mitochondrial membrane is important for determining the efficiency of ATP production via redox reactions. The activity of respiratory chain complexes II, III and IV increased after AP intake, suggesting that the electron transport chain pathway, starting with succinic acid oxidation, was mainly activated.

Epigallocatechin‐3‐gallate (EGCg), an abundant catechin in green tea, increases mitophagy (Takahashi et al., 2017). Takahashi et al. (2017) demonstrated that the LC3‐II/I ratio in EGCg‐treated cage‐control animal muscles was significantly greater (193.3%) than that in vehicle‐treated cage‐control animal muscles. Nogueira et al. (2011) evaluated the endurance of epicatechin‐ and water‐treated mice. They showed that the treadmill endurance capacity of epicatechin‐treated mice was significantly improved compared to that of water‐treated mice. Additionally, epicatechin administration activates citrate synthase activity and mitochondrial biosynthesis (Moreno‐Ulloa et al., 2013). The AP used in the present study contained epicatechin. Moreover, AP ingestion degrades procyanidins in rats to produce epicatechins (Manach et al., 2004; Shoji et al., 2006). Therefore, procyanidins and flavan‐3‐ols may be candidates for increasing TFEB expression and enhancing mitophagy in skeletal muscle. Respiratory chain complex enzyme activity increased mainly in the 0.5% AP group, whereas TFEB and mitophagy‐related factors significantly increased mainly in the 5% AP group. Therefore, it is possible that these increases were due to the different components of the AP. Further studies are required to determine which component of AP is beneficial. It is also necessary to measure endurance capacity using a treadmill during AP and/or component feeding.

In summary, we conclude that AP feeding increases respiratory chain complex enzyme activity in rat skeletal muscles, which may increase ATP synthesis efficiency and improve muscle endurance. Moreover, AP administration increased TFEB activation, and mitophagy may be enhanced to promote dysfunctional mitochondrial degradation.

## AUTHOR CONTRIBUTIONS

Conception or design of the work: Yuki Yoshida, Yuki Tamura, Koichi Nakazato. Acquisition, analysis of data for the work: Yuki Yoshida. Interpretation of data for the work: Yuki Yoshida, Yuki Tamura, Karina Kouzaki, Koichi Nakazato. Drafting of the work or revising it critically for important intellectual content: Yuki Yoshida, Yuki Tamura, Karina Kouzaki, Koichi Nakazato. Preparation of figures and drafting manuscript: Yuki Yoshida. All authors approved the final version of the manuscript. All authors agree to be accountable for all aspects of the work in ensuring that questions related to the accuracy or integrity of any part of the work are appropriately investigated and resolved. All persons designated as authors qualify for authorship, and all those who qualify for authorship are listed.

## CONFLICT OF INTEREST

The authors have no conflicts of interest to declare.

## Supporting information

Statistical Summary Document

## Data Availability

Data will be made available upon request to the authors and a formal data‐sharing agreement.
