# Supplementary material for: Dietary apple polyphenols enhance mitochondrial turnover and respiratory chain enzymes
Source: Exp Physiol. 2023 Sep 1;108(10):1295–307. doi: 10.1113/EP091154 (PMC10988434; doi:10.1113/EP091154)
Supplement: Supplementary file 1 — Statistical Summary Document [file EPH-108-1295-s001.docx]

**Manuscript Title:** Dietary apple polyphenols enhance mitochondrial turnover and respiratory chain enzymes

**Authors:** Yuki Yoshida, Yuki Tamura, Karina Kouzaki & Koichi Nakazato

**Animal model used, if applicable:** Wistar rat

**Underlying hypothesis:** This investigation tested the hypothesis that investigation of apple polyphenols increas mitochondrial biogenesis and promote ATP synthesis in rat skeletal muscle. We think this leads to improvement in endurance capacity.

**Definitions of ‘n’:**

All experiments were conducted the same ‘n’. Number of animals of fed with or without apple polyphenols.

AIN-93M-based normal diet supplemented with 5% apple polyphenols (5% AP group, n=8),

AIN-93M-based normal diet supplemented with 0.5% apple polyphenols (0.5% AP group, n=8),

AIN-93M-based normal diet (control group, n=8)

**Statistical summary table:**

| Experimental question number* | Finding/ conclusion | Experimental location/ variable  e.g. muscle, neocortex or genotype | Mean value  (or other summary statistic) | SD | n val. | P** | Units | Data comparisons  e.g. WT vs KO | Statistical test | Any other variable  e.g. subjects’ age or sex | Figure/ table in which data are presented | Comments  e.g. observation |
| --- | --- | --- | --- | --- | --- | --- | --- | --- | --- | --- | --- | --- |
| 1. Muscle weight (Medial, Lateral and total Gastrocnemius) change by AP administration? | No muscle weigh change observed (Medial, Lateral and total Gastrocnemius) | Medial Gastrocnemius, Control | 1704.6 | 108.7 | 8 | 0.952 | mg | Control Vs 0.5%AP | 1-way ANOVA Fisher‘s PLSD test | METHODS, Animals, experimental diets | 1C |  |
|  |  | Medial Gastrocnemius, 0.5%AP | 1700.7 | 114.1 | 8 | 0.695 |  | 0.5%AP Vs 5%AP |  |  |  |  |
|  |  | Medial Gastrocnemius, 5%AP | 1674.9 | 159.3 | 8 | 0.651 |  | Control Vs 5%AP |  |  |  |  |
|  |  | Lateral Gastrocnemius, Control | 1926.9 | 133.3 | 8 | 0.920 | mg | Control Vs 0.5%AP | 1-way ANOVA Fisher‘s PLSD test |  | 1D |  |
|  |  | Lateral Gastrocnemius, 0.5%AP | 1920.0 | 128.9 | 8 | 0.927 |  | 0.5%AP Vs 5%AP |  |  |  |  |
|  |  | Lateral Gastrocnemius, 5%AP | 1913.8 | 142.9 | 8 | 0.848 |  | Control Vs 5%AP |  |  |  |  |
|  |  | Total Gastrocnemius, Control | 3631.5 | 232.3 | 8 | 0.932 | mg | Control Vs 0.5%AP | 1-way ANOVA Fisher‘s PLSD test |  | 1E |  |
|  |  | Total Gastrocnemius, 0.5%AP | 3620.7 | 227.8 | 8 | 0.800 |  | 0.5%AP Vs 5%AP |  |  |  |  |
|  |  | Total Gastrocnemius, 5%AP | 3588.7 | 285.0 | 8 | 0.735 |  | Control Vs 5%AP |  |  |  |  |
| 2. Western blot and enzyme activity, cytochrome c and citrate synthase activity in gastrocnemius change by AP administration? | 5%AP high in cytochrome c, 5%AP and 0.5%AP high in citrate synthase activity | cytocrome c, Control | 1 | 0.38 | 8 | 0.889 | fold | Control Vs 0.5%AP | 1-way ANOVA Fisher‘s PLSD test |  | 2B |  |
|  |  | cytocrome c, 0.5%AP | 1.01 | 0.31 | 8 | 0.058 |  | 0.5%AP Vs 5%AP |  |  |  |  |
|  |  | cytocrome c, 5%AP | 1.33 | 0.37 | 8 | **0.044** |  | Control Vs 5%AP |  |  |  |  |
|  |  | Citrate Synthase activity, Control | 12.86 | 0.57 | 8 | 0.113 | Activity (mmol/min/μg) | Control Vs 0.5%AP | 1-way ANOVA Fisher‘s PLSD test |  | 2C |  |
|  |  | Citrate Synthase activity, 0.5%AP | 13.53 | 1.09 | 8 | **0.014** |  | 0.5%AP Vs 5%AP |  |  |  |  |
|  |  | Citrate Synthase activity, 5%AP | 14.61 | 0.68 | 8 | **0.0003** |  | Control Vs 5%AP |  |  |  |  |
| 3. Western blot, Mitochondrial biogenesis in gastrocnemius (PGC1α, TFEB and TFE3) change by AP administration? | 5%AP high in TFEB and nuclear TFEB | PGC1α, Control | 1 | 0.17 | 8 | 0.161 | fold | Control Vs 0.5%AP | 1-way ANOVA Fisher‘s PLSD test |  | 3D |  |
|  |  | PGC1α, 0.5%AP | 0.90 | 0.14 | 8 | 0.815 |  | 0.5%AP Vs 5%AP |  |  |  |  |
|  |  | PGC1α, 5%AP | 0.91 | 0.12 | 8 | 0.238 |  | Control Vs 5%AP |  |  |  |  |
|  |  | TFEB, Control | 1 | 0.13 | 8 | 0.734 | fold | Control Vs 0.5%AP | 1-way ANOVA Fisher‘s PLSD test |  | 3E |  |
|  |  | TFEB, 0.5%AP | 1.03 | 0.23 | 8 | 0.086 |  | 0.5%AP Vs 5%AP |  |  |  |  |
|  |  | TFEB, 5%AP | 1.18 | 0.12 | 8 | **0.044** |  | Control Vs 5%AP |  |  |  |  |
|  |  | TFE3, Control | 1 | 0.14 | 8 | 0.558 | fold | Control Vs 0.5%AP | 1-way ANOVA Fisher‘s PLSD test |  | 3F |  |
|  |  | TFE3, 0.5%AP | 1.04 | 0.12 | 8 | 0.614 |  | 0.5%AP Vs 5%AP |  |  |  |  |
|  |  | TFE3, 5%AP | 1.01 | 0.16 | 8 | 0.935 |  | Control Vs 5%AP |  |  |  |  |
|  |  | nuclear PGC-1α, Control | 1 | 0.16 | 8 | 0.573 | fold | Control Vs 0.5%AP | 1-way ANOVA Fisher‘s PLSD test |  | 3G |  |
|  |  | nuclear PGC-1α, 0.5%AP | 1.05 | 0.17 | 8 | 0.551 |  | 0.5%AP Vs 5%AP |  |  |  |  |
|  |  | nuclear PGC-1α, 5%AP | 1.10 | 0.18 | 8 | 0.252 |  | Control Vs 5%AP |  |  |  |  |
|  |  | nuclear TFEB, Control | 1 | 0.18 | 8 | 0.697 | fold | Control Vs 0.5%AP | 1-way ANOVA Fisher‘s PLSD test |  | 3H |  |
|  |  | nuclear TFEB, 0.5%AP | 1.04 | 0.19 | 8 | **0.014** |  | 0.5%AP Vs 5%AP |  |  |  |  |
|  |  | nuclear TFEB, 5%AP | 1.30 | 0.21 | 8 | **0.006** |  | Control Vs 5%AP |  |  |  |  |
|  |  | nuclear TFE3, Control | 1 | 0.14 | 8 | 0.171 | fold | Control Vs 0.5%AP | 1-way ANOVA Fisher‘s PLSD test |  | 3I |  |
|  |  | nuclear TFE3, 0.5%AP | 1.11 | 0.15 | 8 | 0.506 |  | 0.5%AP Vs 5%AP |  |  |  |  |
|  |  | nuclear TFE3, 5%AP | 1.06 | 0.17 | 8 | 0.468 |  | Control Vs 5%AP |  |  |  |  |
| 4. Western blot and qRT-PCR,  Mitochondrial contents in gastrocnemius change by AP administration? | No change observed | VDAC, Control | 1 | 0.23 | 8 | 0.990 | fold | Control Vs 0.5%AP | 1-way ANOVA Fisher‘s PLSD test |  | 4B |  |
|  |  | VDAC, 0.5%AP | 1.00 | 0.22 | 8 | 0.961 |  | 0.5%AP Vs 5%AP |  |  |  |  |
|  |  | VDAC, 5%AP | 1.01 | 0.19 | 8 | 0.950 |  | Control Vs 5%AP |  |  |  |  |
|  |  | CI-NDUFB8, Control | 1 | 0.48 | 8 | 0.651 | fold | Control Vs 0.5%AP | 1-way ANOVA Fisher‘s PLSD test |  | 4C |  |
|  |  | CI-NDUFB8, 0.5%AP | 0.85 | 0.62 | 8 | 0.844 |  | 0.5%AP Vs 5%AP |  |  |  |  |
|  |  | CI-NDUFB8, 5%AP | 0.92 | 0.79 | 8 | 0.798 |  | Control Vs 5%AP |  |  | 4C |  |
|  |  | CII-SDHB, Control | 1 | 0.34 | 8 | 0.738 | fold | Control Vs 0.5%AP | 1-way ANOVA Fisher‘s PLSD test |  |  |  |
|  |  | CII-SDHB, 0.5%AP | 0.93 | 0.39 | 8 | 0.768 |  | 0.5%AP Vs 5%AP |  |  |  |  |
|  |  | CII-SDHB, 5%AP | 0.99 | 0.47 | 8 | 0.968 |  | Control Vs 5%AP |  |  |  |  |
|  |  | CIV-MTCO1, Control | 1 | 0.24 | 8 | 0.162 | fold | Control Vs 0.5%AP | 1-way ANOVA Fisher‘s PLSD test |  |  |  |
|  |  | CIV-MTCO1, 0.5%AP | 0.83 | 0.27 | 8 | 0.371 |  | 0.5%AP Vs 5%AP |  |  |  |  |
|  |  | CIV-MTCO1, 5%AP | 0.94 | 0.20 | 8 | 0.597 |  | Control Vs 5%AP |  |  |  |  |
|  |  | CIII-UQCRC, Control | 1 | 0.14 | 8 | 0.767 | fold | Control Vs 0.5%AP | 1-way ANOVA Fisher‘s PLSD test |  |  |  |
|  |  | CIII-UQCRC, 0.5%AP | 0.98 | 0.14 | 8 | 0.428 |  | 0.5%AP Vs 5%AP |  |  |  |  |
|  |  | CIII-UQCRC, 5%AP | 0.93 | 0.12 | 8 | 0.280 |  | Control Vs 5%AP |  |  |  |  |
|  |  | CV-ATP5A, Control | 1 | 0.08 | 8 | 0.793 | fold | Control Vs 0.5%AP | 1-way ANOVA Fisher‘s PLSD test |  |  |  |
|  |  | CV-ATP5A, 0.5%AP | 0.99 | 0.08 | 8 | 0.620 |  | 0.5%AP Vs 5%AP |  |  |  |  |
|  |  | CV-ATP5A, 5%AP | 1.01 | 0.09 | 8 | 0.815 |  | Control Vs 5%AP |  |  |  |  |
|  |  | 16s rRNA / β-Actin, Control | 1 | 0.38 | 8 | 0.582 | fold | Control Vs 0.5%AP | 1-way ANOVA Fisher‘s PLSD test |  | 4D |  |
|  |  | 16s rRNA / β-Actin, 0.5%AP | 1.15 | 0.63 | 8 | 0.562 |  | 0.5%AP Vs 5%AP |  |  |  |  |
|  |  | 16s rRNA / β-Actin, 5%AP | 0.99 | 0.61 | 8 | 0.976 |  | Control Vs 5%AP |  |  |  |  |
|  |  | tRNA / GAPDH, Control | 1 | 0.21 | 8 | 0.796 | fold | Control Vs 0.5%AP | 1-way ANOVA Fisher‘s PLSD test |  | 4E |  |
|  |  | tRNA / GAPDH, 0.5%AP | 0.97 | 0.26 | 8 | 0.332 |  | 0.5%AP Vs 5%AP |  |  |  |  |
|  |  | tRNA / GAPDH, 5%AP | 0.85 | 0.25 | 8 | 0.223 |  | Control Vs 5%AP |  |  |  |  |
| 5. Western blot, Effect of Mitochondrial protein quality control in gastrocnemius by AP administration?. | No change observed | HSP60, Control | 1 | 0.23 | 8 | 0.427 | fold | Control Vs 0.5%AP | 1-way ANOVA Fisher‘s PLSD test |  | 5B |  |
|  |  | HSP60, 0.5%AP | 0.93 | 0.15 | 8 | 0.273 |  | 0.5%AP Vs 5%AP |  |  |  |  |
|  |  | HSP60, 5%AP | 1.03 | 0.09 | 8 | 0.765 |  | Control Vs 5%AP |  |  |  |  |
|  |  | mtHSP70, Control | 1 | 0.36 | 8 | 0.568 | fold | Control Vs 0.5%AP | 1-way ANOVA Fisher‘s PLSD test |  | 5C |  |
|  |  | mtHSP70, 0.5%AP | 1.08 | 0.22 | 8 | 0.717 |  | 0.5%AP Vs 5%AP |  |  |  |  |
|  |  | mtHSP70, 5%AP | 1.03 | 0.18 | 8 | 0.833 |  | Control Vs 5%AP |  |  |  |  |
|  |  | ClpP, Control | 1 | 0.31 | 8 | 0.580 | fold | Control Vs 0.5%AP | 1-way ANOVA Fisher‘s PLSD test |  | 5D |  |
|  |  | ClpP, 0.5%AP | 0.92 | 0.25 | 8 | 0.410 |  | 0.5%AP Vs 5%AP |  |  |  |  |
|  |  | ClpP, 5%AP | 1.04 | 0.25 | 8 | 0.783 |  | Control Vs 5%AP |  |  |  |  |
| 6. Western blot (mitochondria fraction), Effect of mitochondrial autophagy in gastrocnemius by AP administration? | 5%AP high in LC3B-II | LC3B-I, Control (gastrocnemius hole) | 1 | 0.32 | 8 | 0.799 | fold | Control Vs 0.5%AP | 1-way ANOVA Fisher‘s PLSD test |  | 6B |  |
|  |  | LC3B-I, 0.5%AP(gastrocnemius hole) | 1.03 | 0.20 | 8 | 0.354 |  | 0.5%AP Vs 5%AP |  |  |  |  |
|  |  | LC3B-I, 5%AP(gastrocnemius hole) | 0.15 | 0.18 | 8 | 0.242 |  | Control Vs 5%AP |  |  |  |  |
|  |  | LC3B-II, Control(gastrocnemius hole) | 1 | 0.30 | 8 | 0.145 | fold | Control Vs 0.5%AP | 1-way ANOVA Fisher‘s PLSD test |  | 6C |  |
|  |  | LC3B-II, 0.5%AP(gastrocnemius hole) | 1.20 | 0.16 | 8 | 0.110 |  | 0.5%AP Vs 5%AP |  |  |  |  |
|  |  | LC3B-II, 5%AP(gastrocnemius hole) | 1.41 | 0.29 | 8 | **0.005** |  | Control Vs 5%AP |  |  |  |  |
|  |  | LC3B-II/I, Control(gastrocnemius hole) | 1.01 | 0.18 | 8 | 0.114 | fold | Control Vs 0.5%AP | 1-way ANOVA Fisher‘s PLSD test |  | 6D |  |
|  |  | LC3B-II/I, 0.5%AP(gastrocnemius hole) | 1.18 | 0.18 | 8 | 0.653 |  | 0.5%AP Vs 5%AP |  |  |  |  |
|  |  | LC3B-II/I, 5%AP(gastrocnemius hole) | 1.23 | 0.24 | 8 | **0.047** |  | Control Vs 5%AP |  |  |  |  |
|  | 5%AP and 0.5%AP high in LC3B | LC3B-II, Control | 1 | 0.27 | 8 | **0.042** | fold | Control Vs 0.5%AP | 1-way ANOVA Fisher‘s PLSD test |  | 6F |  |
|  |  | LC3B-II, 0.5%AP | 1.26 | 0.24 | 8 | 0.969 |  | 0.5%AP Vs 5%AP |  |  |  |  |
|  |  | LC3B-II, 5%AP | 1.27 | 0.23 | 8 | **0.039** |  | Control Vs 5%AP |  |  |  |  |
|  |  | Ubiquitin, Control | 1 | 0.21 | 8 | 0.454 | fold | Control Vs 0.5%AP | 1-way ANOVA Fisher‘s PLSD test |  | 6G |  |
|  |  | Ubiquitin, 0.5%AP | 1.17 | 0.36 | 8 | 0.280 |  | 0.5%AP Vs 5%AP |  |  |  |  |
|  |  | Ubiquitin, 5%AP | 1.28 | 0.27 | 8 | 0.051 |  | Control Vs 5%AP |  |  |  |  |
| 7. Effect of enzyme activity of mitochondrial respiratory chain complex in the gastrocnemius by AP administration? | 0.5%AP high in Complex II+III and Complex IV, 5%AP high in Complex II+III | Complex I, Control | 8.22 | 1.41 | 8 | 0.876 | Activity (mmol/min/μg) | Control Vs 0.5%AP | 1-way ANOVA Fisher‘s PLSD test |  | 7A |  |
|  |  | Complex I, 0.5%AP | 9.30 | 1.85 | 8 | 0.876 |  | 0.5%AP Vs 5%AP |  |  |  |  |
|  |  | Complex I, 5%AP | 8.74 | 1.95 | 8 | 0.876 |  | Control Vs 5%AP |  |  |  |  |
|  |  | Complex II, Control | 4.27 | 1.24 | 8 | 0.093 | Activity (mmol/min/μg) | Control Vs 0.5%AP | 1-way ANOVA Fisher‘s PLSD test |  | 7B |  |
|  |  | Complex II, 0.5%AP | 5.33 | 1.45 | 8 | 0.773 |  | 0.5%AP Vs 5%AP |  |  |  |  |
|  |  | Complex II, 5%AP | 5.16 | 0.89 | 8 | 0.157 |  | Control Vs 5%AP |  |  |  |  |
|  |  | Complex I+III, Control | 4.86 | 1.04 | 8 | 0.187 | Activity (mmol/min/μg) | Control Vs 0.5%AP | 1-way ANOVA Fisher‘s PLSD test |  | 7C |  |
|  |  | Complex I+III, 0.5%AP | 5.72 | 0.83 | 8 | 0.785 |  | 0.5%AP Vs 5%AP |  |  |  |  |
|  |  | Complex I+III, 5%AP | 5.54 | 1.71 | 8 | 0.289 |  | Control Vs 5%AP |  |  |  |  |
|  |  | Complex II+III, Control | 0.75 | 0.21 | 8 | **0.012** | Activity (mmol/min/μg) | Control Vs 0.5%AP | 1-way ANOVA Fisher‘s PLSD test |  | 7D |  |
|  |  | Complex II+III, 0.5%AP | 1.22 | 0.40 | 8 | 0.730 |  | 0.5%AP Vs 5%AP |  |  |  |  |
|  |  | Complex II+III, 5%AP | 1.16 | 0.38 | 8 | **0.026** |  | Control Vs 5%AP |  |  |  |  |
|  |  | Complex IV, Control | 1.96 | 0.33 | 8 | **0.035** | Activity (mmol/min/μg) | Control Vs 0.5%AP | 1-way ANOVA Fisher‘s PLSD test |  | 7E |  |
|  |  | Complex IV, 0.5%AP | 2.42 | 0.31 | 8 | 0.586 |  | 0.5%AP Vs 5%AP |  |  |  |  |
|  |  | Complex IV, 5%AP | 2.31 | 0.57 | 8 | 0.103 |  | Control Vs 5%AP |  |  |  |  |
|  |  |  |  |  |  |  |  |  |  |  |  |  |

*You may use multiple lines for the same question to indicate multiple comparisons

** Authors may wish to make the text bold where p is considered significant against a stated confidence limit.
